# Gender benefit in laparoscopic surgical performance using a 3D-display system: data from a randomized cross-over trial

**DOI:** 10.1007/s00464-021-08785-4

**Published:** 2021-11-08

**Authors:** Jana Busshoff, Rabi R. Datta, Thomas Bruns, Robert Kleinert, Bernd Morgenstern, David Pfister, Costanza Chiapponi, Hans F. Fuchs, Michael Thomas, Caroline Gietzelt, Andrea Hedergott, Desdemona Möller, Martin Hellmich, Christiane J. Bruns, Dirk L. Stippel, Roger Wahba

**Affiliations:** 1 Department of General, Visceral, Cancer and Transplantation Surgery, Faculty of Medicine and University Hospital of Cologne, University of Cologne, Joseph-Stelzmann-Straße 9, 50931 Cologne, Germany; 2 Department of Obstetrics and Gynecology, Faculty of Medicine and University Hospital of Cologne, University of Cologne, Cologne, Germany; 3 Department of General and Visceral Surgery, Protestant Hospital of Bethel Foundation, University Hospital OWL of the University of Bielefeld, Bielefeld, Germany; 4 Department of Urology, Faculty of Medicine and University Hospital of Cologne, University of Cologne, Cologne, Germany; 5 Department of Ophthalmology, Faculty of Medicine and University Hospital of Cologne, University of Cologne, Cologne, Germany; 6 Department of Business Administration and Health Care Management, Faculty of Management, Economics and Social Sciences, University of Cologne, Cologne, Germany; 7 Institute of Medical Statistics and Computational Biology, Faculty of Medicine and University Hospital Cologne, University of Cologne, Cologne, Germany

**Keywords:** Gender, Laparoscopic surgery, Medical education, 3D laparoscopy

## Abstract

**Background:**

The use of 3D technique compared to high-resolution 2D-4K-display technique has been shown to optimize spatial orientation and surgical performance in laparoscopic surgery. Since women make up an increasing amount of medical students and surgeons, this study was designed to investigate whether one gender has a greater benefit from using a 3D compared to a 4K-display system.

**Methods:**

In a randomized cross-over trial, the surgical performance of male and female medical students (MS), non-board certified surgeons (NBCS), and board certified surgeons (BCS) was compared using 3D- vs. 4K-display technique at a minimally invasive training parkour with multiple surgical tasks and repetitions.

**Results:**

128 participants (56 women, 72 men) were included. Overall parkour time in seconds was 3D vs. 4K for all women 770.7 ± 31.9 vs. 1068.1 ± 50.0 (*p* < 0.001) and all men 664.5 ± 19.9 vs. 889.7 ± 31.2 (*p* < 0.001). Regarding overall mistakes, participants tend to commit less mistakes while using the 3D-vision system, showing 10.2 ± 1.1 vs. 13.3 ± 1.3 (*p* = 0.005) for all women and 9.6 ± 0.7 vs. 12.2 ± 1.0 (*p* = 0.001) for all men. The benefit of using a 3D system, measured by the difference in seconds, was for women 297.3 ± 41.8 (27.84%) vs. 225.2 ± 23.3 (25.31%) for men (*p* = 0.005). This can be confirmed in the MS group with 327.6 ± 65.5 (35.82%) vs. 249.8 ± 33.7 (32.12%), *p* = 0.041 and in the NBCS group 359 ± 52.4 (28.25%) vs. 198.2 ± 54.2 (18.62%), *p* = 0.003. There was no significant difference in the BCS group.

**Conclusion:**

3D laparoscopic display technique optimizes surgical performance compared to the 2D-4K technique for both women and men. The greatest 3D benefit was found for women with less surgical experience. As a possible result of surgical education, this gender specific difference disappears with higher grade of experience. Using a 3D-vision system could facilitate surgical apprenticeship, especially for women.

For 2 decades, the number of women studying medicine steadily increases, in Germany currently more than 50% of medical students are female [1–3]. Although the number of female surgeons rises compared to their male colleagues, only a minority of women decide on a career in surgery [2, 4, 5].

Minimally invasive surgery (MIS) is an important component of modern abdominal surgery. During the last years, more and more complex procedures were performed laparoscopically. This evolution is supported by modern medical devices and the display systems used in the operation theater [6]. It was demonstrated that 3D laparoscopic vision systems optimize surgical performance compared to the high-resolution 2D-4 K technique, and that surgeons benefit from the enhanced vision system, regardless of their level of experience [7–9].

It is debated whether women and men are processing visual impressions in neurophysiologically different ways [10–16]. Men seem to have greater sensitivity for rapidly moving stimuli and their brains might be structured to connect between perception and coordinated action while female brains tend to promote communication between processing of analytical and intuitive impressions [10, 13]. Consequently, there is a difference in relation to learning from visual impressions or solving visually demanding tasks, as given in laparoscopic surgery [17, 18]. In tasks concerning navigation or spatial orientation, men seem to perform better [19, 20]. Studies comparing surgical performance in laparoscopy of women versus men is rare and inconclusive [21–28]. Recent studies comparing 3D- vs. conventional 2D- or modern 2D-4K-vision systems did neither comment on participants’ gender nor the correlation between gender and vision system used [8, 9, 29–33]. Data concerning gender specific benefits regarding the use of a 3D-vision system does not exist. Therefore, the aim of this study was to analyze whether surgical performance is different between genders and whether one gender has a greater benefit from using a 3D laparoscopic visualization system compared to a high-resolution (4 K) 2D system.

## Methods

The lead investigators and a statistician planned this investigator-initiated trial as randomized cross-over, single-blinded trial. Approval was obtained from the Ethics Committee of the University Hospital of Cologne (No. 17-388). The study protocol was registered at clinicaltrial.gov (NCT03445429) and published upfront [34]. The initial results of the trial have also been published [7]. Participants gave written consent and were randomized by an independent data trustee. Data were collected and analyzed by the authors.

### Participants

Participation was voluntary. Medical students (MS) came from the University of Cologne. Board (BCS) and Non-Board (NBCS) certified surgeons were working in the Department of General, Visceral, Cancer and Transplantation Surgery, Gynecology and Urology at University Hospital of Cologne and nine affiliated hospitals of the Cologne area.

Excluded from the trial were MS with any experience in laparoscopy, subjects with general experience in the minimally invasive training Parkour, non-correctable vision disorders, known impaired stereoscopic vision or manual skill disorders.

### Study design

This trial compared surgical performance of men and women at a minimally invasive training course using a 3D- and a 2D-4K-display system technique. The participants (MS, NBCS, BCS) were randomized to start the parkour in 3D-display system followed by a second turn with 4K or the other way around. Each turn included 7 tasks (5 for MS) of different demands. The tasks were called “rope pass”, “paper cut”, “pegboard transfer” (FLS), “needle threading”, “needle recapping”, “circle cutting” (FLS), and “knot tying” (FLS).

The laparoscopic performance was documented by 2D-video, which was evaluated afterward by two blinded independent investigators independently. The passive polarizing 3D laparoscopic system “Einstein Vision® 2.0” (10 mm 30° camera, 3D full high-definition 32 “ monitor, Aesculap AG, Tuttlingen, Germany) and the 2D-4K System “Visera 4K Ultra High Definition” (10 mm 30° camera, 55” monitor, Olympus Medical system Olympus Europa SE & Co. KG, Hamburg, Germany) were used. After the parkour an ophthalmological evaluation of the subjects with multiple tests for stereoscopic vision followed.

### Outcome parameters

The primary outcome parameter was surgical performance, measured by “time in seconds” and “number of mistakes”, comparing results in 3D and 2D-4K for men and women. A mistake was defined as deviation from perfect performance as detailed described in the study protocol [7].

Secondary outcome parameter was the quantification of the differences in between display systems between male and female gender.

### Statistical analysis

From preliminary experiments of the study group and published data a standardized effect of 0.5 in favor of the 3D system was expected. A sample size of 34 per stratum is required to detect this standardized effect of 0.5 with a power of 80% at 2-sided type I error 5% [7]. Quantitative variables are summarized by valid count (*n*), mean ± standard deviation, qualitative variables by absolute and relative frequencies (percentages). Outcome measures are evaluated by linear mixed models for repeated measures (MMRM) with main effects experience, sequence, method, repetition, and interactions (type III SS, REML, heterogeneous compound symmetry covariance matrix). Estimated marginal means and contrasts were derived. To ensure convergence of algorithms and to get readily interpretable results, separate models were fitted by gender and the obtained marginal means and contrasts were compared based on reported standard errors and degrees of freedom. Two-sided p values < 0.05 were interpreted to indicate statistical significance. Statistical data analysis was performed using SPSS Statistics 26 (IBM Corp., Armonk, NY, USA).

## Results

Between February 2018 and October 2019, 133 subjects were randomized, 128 of which could be included in the final analysis. This cohort included 56 women, corresponding to a share of 44%. In MS group it was a 42% female share (*n* = 21), for NBCS 72% (*n* = 28) and for BCS 18% (*n* = 7). Interrater reliability between the two rater was given. 74% of the subjects underwent ophthalmological evaluation, showing in 95% normal stereoscopic vision without gender specific differences.

### Comparison of female vs. male surgeons performing the minimally invasive training course

Table 1 shows mean overall parkour time including time required for all tasks, averaged from both display systems comparing female vs. male participants, was 920 ± 31.4 vs. 776.9 ± 27 (*p* = 0.001).

**Table 1:** Surgical performance comparing 3D- vs. 4 K-display system

		Rope pass			Paper cut			Pegboard transfer		
		3D	4 K	*p*	3D	4 K	*p*	3D	4 K	*p*
All	f (*n* = 56)	60.6 ± 2.5	89.6 ± 4.5	< 0.001	116.9 ± 9.2	149.3 ± 12.4	0.001	109.6 ± 4.1	143.5 ± 6.4	< 0.001
	m (*n* = 71)	55.2 ± 1.9	77.7 ± 2.7	< 0.001	102.1 ± 6.4	118.7 ± 7.7	0.003	97.1 ± 1.8	136.4 ± 3.1	< 0.001
	p f vs m			0.019			0.044			0.043
MS	f (*n* = 21)	78.7 ± 3.2	122.3 ± 5.3	< 0.001	140.1 ± 12.3	184.4 ± 15.9	0.005	124.8 ± 4.3	174.3 ± 6.9	< 0.001
	m (*n* = 27)	73.9 ± 2.8	116.5 ± 4.7	< 0.001	129.4 ± 10.1	158.3 ± 13	< 0.001	112.0 ± 3.7	168.0 ± 6.1	< 0.001
	p f vs m			0.297			0.265			0.158
NBCS	f (*n* = 28)	50.7 ± 2.8	73.8 ± 4.6	< 0.001	112.2 ± 9.7	141.9 ± 12.3	0.008	106.7 ± 3.7	135.9 ± 6.0	< 0.001
	m (*n* = 11)	50.2 ± 4.5	61.3 ± 7.4	0.020	100.5 ± 15.6	95.5 ± 20.1	0.304	101.2 ± 5.9	136.4 ± 9.6	< 0.001
	p f vs m			0.296			0.132			0.758
BCS	f (*n* = 7)	52.5 ± 5.6	72.6 ± 9.3	0.035	99 ± 19.8	122.0 ± 24.8	< 0.001	97.2 ± 7.4	120.4 ± 12.1	0.045
	m (*n* = 33)	41.6 ± 2.6	55.4 ± 4.2	< 0.001	76.5 ± 9.1	101.8 ± 11.4	0.001	78.2 ± 3.4	104.8 ± 5.5	< 0.001
	p f vs m			0.057			0.342			0.077

		Needle threading			Needle recapping			Circle cutting		
		3D	4 K	*p*	3D	4 K	*p*	3D	4 K	*p*
all	f (*n* = 56)	111.0 ± 13.9	237.4 ± 24.5	< 0.001	93.2 ± 11.7	129.0 ± 14.3	0.017	135.5 ± 9.3	146.4 ± 12.8	0.162
	m (*n* = 71)	84.6 ± 7.1	170.4 ± 12.8	< 0.001	78.9 ± 5.9	106.3 ± 7.4	0.001	125.4 ± 6.8	142.6 ± 8.5	< 0.001
	p f vs m			0.009			0.096			0.582
MS	f (*n* = 21)	145.8 ± 15.4	308.3 ± 27.6	< 0.001	113.2 ± 12.8	162.3 ± 15.9	0.018	ND	ND	
	m (*n* = 27)	118.2 ± 13.4	218.6 ± 24.1	< 0.001	94.3 ± 11.2	144.6 ± 14.2	< 0.001	ND	ND	
	p f vs m			0.019			0.239			
NBCS	f (*n* = 28)	107.4 ± 13.2	223.4 ± 23.7	< 0.001	103.6 ± 11.3	145.2 ± 13.7	0.021	144.0 ± 7.8	158.6 ± 10.4	0.036
	m (n = 11)	81.6 ± 21.1	185.7 ± 36.9	< 0.001	86.4 ± 17.7	89.9 ± 21.8	0.859	144.3 ± 12.4	151.9 ± 16.4	0.279
	p f vs m			0.287			0.058			0.846
BCS	f (*n* = 7)	79.6 ± 26.7	179.0 ± 46.6	0.055	62.6 ± 22.3	79.5 ± 27.6	0.634	127.0 ± 15.6	134.1 ± 20.6	0.607
	m (*n* = 33)	53.6 ± 12.2	107.1 ± 21.3	0.001	55.9 ± 10.2	84.5 ± 12.6	0.010	106.5 ± 7.1	133.2 ± 9.4	< 0.001
	p f vs m			0.163			0.970			0.577

		Knot tying			Overall					
		3D	4 K	*p*	3D	4 K	*p*	Mean averaged parkourtime		
All	f (*n* = 56)	310.2 ± 25.1	382 ± 31.1	0.003	770.7 ± 31.9	1068.1 ± 50.0	< 0.001	920.0 ± 31.4		
	m (*n* = 71)	248.9 ± 13.4	329.5 ± 20.3	< 0.001	664.5 ± 19.9	889.7 ± 31.2	< 0.001	776.9 ± 27.0		
	p f vs m			0.053			0.001			
MS	f (*n* = 21)	ND	ND		591.2 ± 40.3	921.1 ± 63.5	< 0.001	756.2 ± 46.2		
	m (*n* = 27)	ND	ND		526.2 ± 32.6	775.2 ± 52.4	< 0.001	650.7 ± 38.2		
	p f vs m						0.082			
NBCS	f (*n* = 28)	322.2 ± 19.0	455.2 ± 25.0	< 0.001	910.4 ± 33.4	1268.9 ± 52.2	< 0.001	1089.7 ± 38.5		
	m (*n* = 11)	303.0 ± 30.7	385.0 ± 40.5	0.001	860.3 ± 53.3	1057.1 ± 85.9	< 0.001	958.7 ± 62.8		
	p f vs m			0.252			0.079			
BCS	f (*n* = 7)	297.7 ± 36.4	309.4 ± 47.9	0.785	814.6 ± 63.9	1014.1 ± 96.4	0.047	914.3 ± 72.6		
	m (*n* = 33)	194.9 ± 16.5	273.5 ± 22.1	< 0.001	607.4 ± 29.6	835.5 ± 45.2	< 0.001	721.4 ± 33.7		
	p f vs m			0.111			0.018	0.018		

It shows performance time in seconds of each task of the minimally invasive training parkour for medicine students, non-board certified surgeons and board certified surgeons comparing 3D vs. 4K as well as female and male participants

*3D* 3-dimensional-display system, *4 K* 2D-4K ultra-high definition-display system, *f* female, *m* male, *MS* medicine students, *NBCS* non-board certified surgeons, *BCS* board certified surgeons, *ND* not done according to study protocol, *p* *p* value

**Fig. 1:**
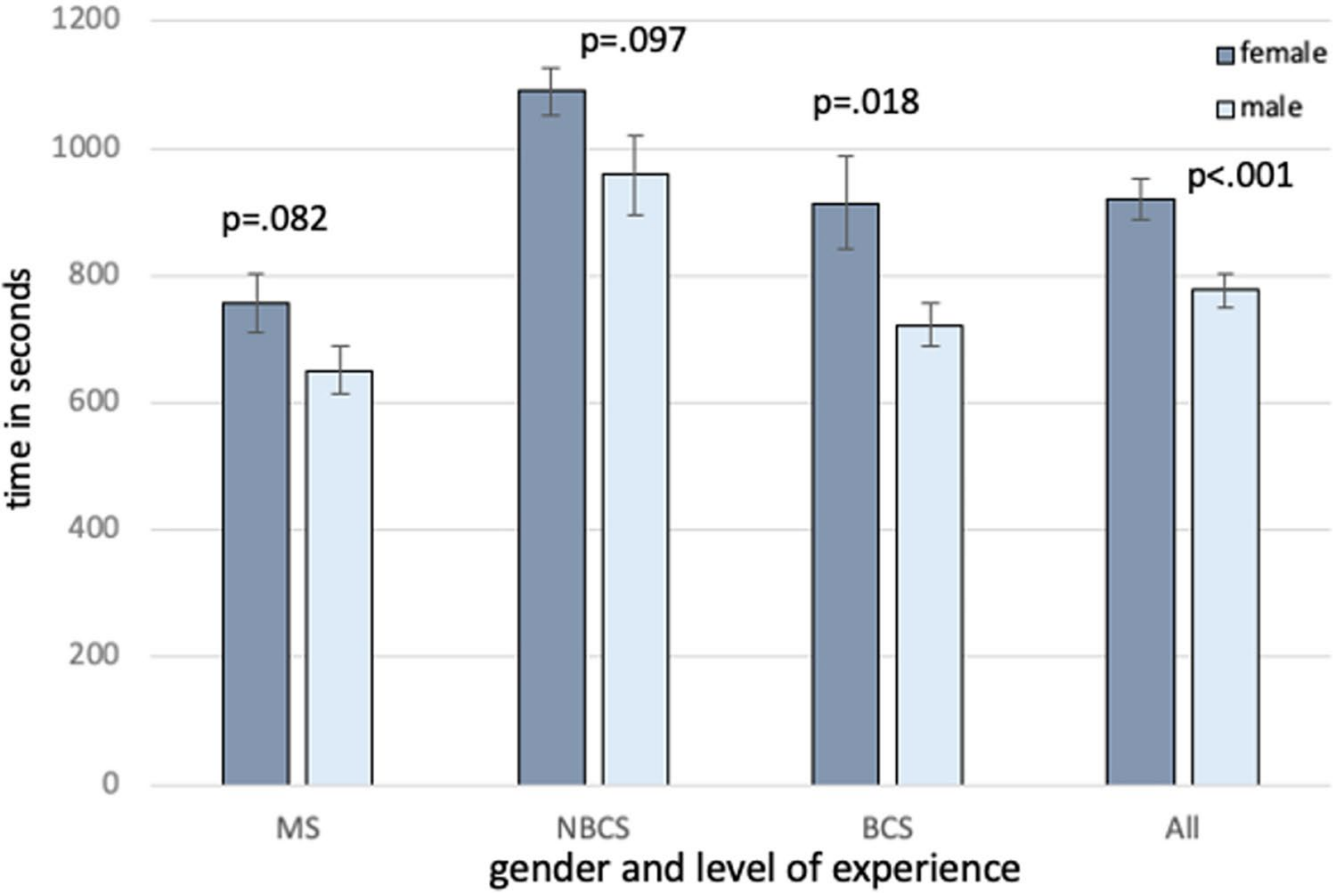
Overall parkour time comparing male and female participants. It shows the overall parkour time (all tasks, mean time averaged from 3D and 4K system) at the minimally invasive training parkour—medicine students (5 tasks), non-board certified surgeons (7 tasks), and board certified surgeons (7 tasks) comparing male and female participants. The overall performance time was significantly shorter in male surgeons regarding BCS and all participants. *MS* medicine students, *NBCS* non-board certified surgeons, *BCS* board certified surgeons, *p* *p* value

For BCS, mean overall performance time for female vs. male was 914.3 ± 72.6 vs. 721.4 ± 33.7 (*p* = 0.018), in MS and NBCS results and did not differ significantly, as shown in Fig. 1.

Comparing overall mistakes of female 11.7 ± 1.0 vs. male 10.9 ± 0.9, rate of mistakes did not show significant differences, neither regarding the subgroups nor all participants combined.

### Comparison of overall performance (time and errors) in female vs. male surgeons using a 3D- vs. 2D-4K-display system

**Fig. 2:**
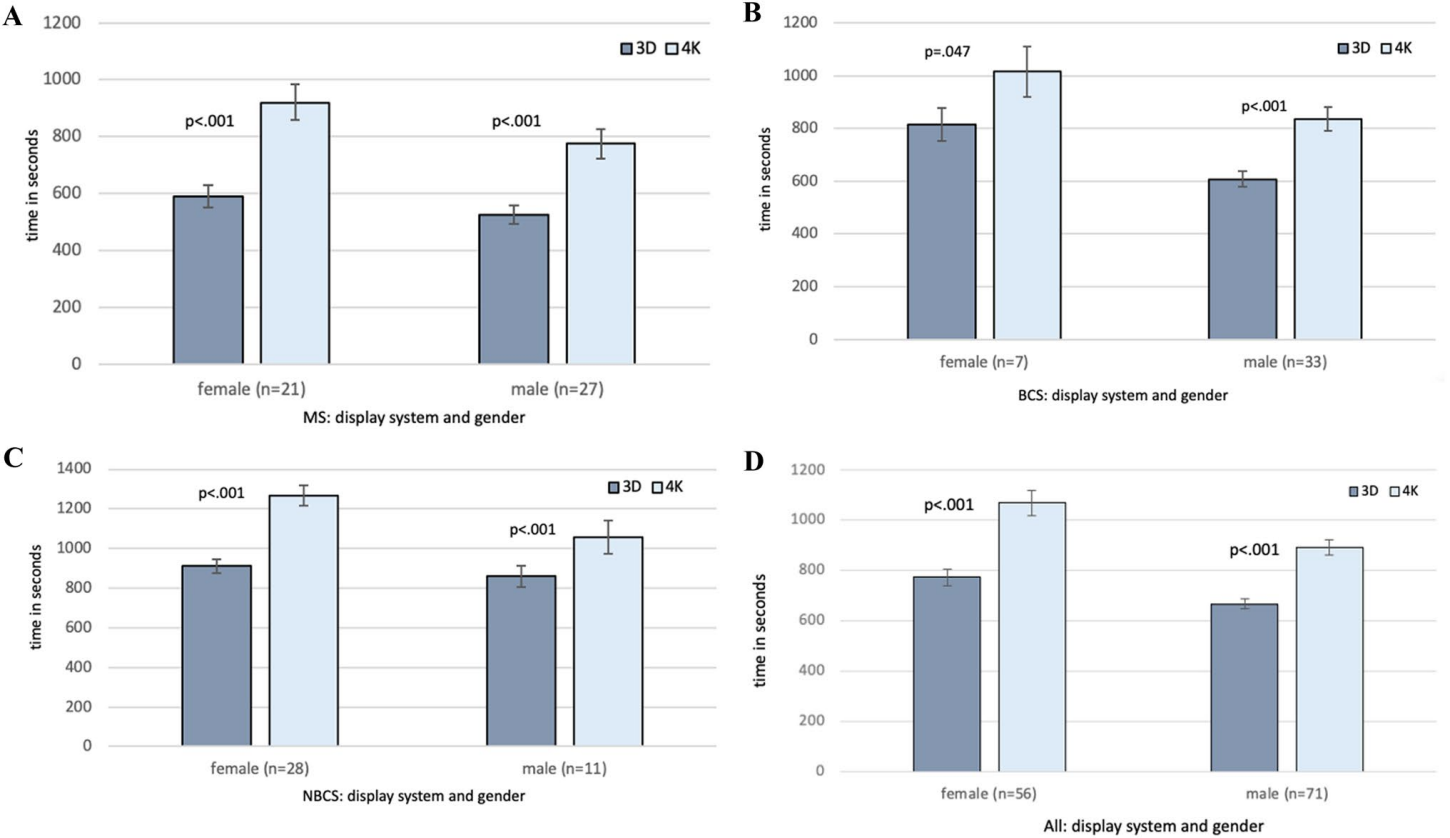
Overall parkour time comparing 3D vs. 4K. It shows overall parkour time (all tasks performed with one display system) comparing 3D- versus 4K-display system at the minimally invasive training parkour in male and female participants. **A** Showing results for all participants, **B** for medicine students, **C** for non-board certified surgeons, and **D** for board certified surgeons. The overall performance time was significantly shorter using the 3D-display system compared to the 4 K-display system for all levels of experience and both gender. *MS* medicine students, *NBCS* non-board certified surgeons, *BCS* board certified surgeons, *p* *p* value, *3D* 3-dimensional-display system, *4 K* 2D-4 K ultra-high definition-display system

Overall performance time comparing 3D- vs. 4K-vision system for females was 770.7 ± 31.9 (3D) vs. 1068.1 ± 50 (4K) (*p* < 0.001) and for men 664.5 ± 19.9 (3D) vs. 889.7 ± 31.2 (4K) (*p* < 0.001) as shown in Fig. 2D. In female MS it was 591.2 ± 40.3 vs. 921.1 ± 63.5 (*p* < 0.001) and in male MS it was 526.2 ± 32.6 vs. 775.2 ± 52.4 (*p* < 0.001) (Fig. 2A). In female NBCS it was 910.4 ± 33.4 vs. 1268.9 ± 52.2 (*p* < 0.001) and in male NBCS it was 860.3 ± 53.3 vs. 1057.1 ± 85.9 (*p* < 0.001) (Fig. 2B). In female BCS it was 814.6 ± 63.9 (3D) vs. 1014.1 ± 96.4 (4 K) (*p* = 0.047) and in male BCS it was 607.4 ± 29.6 vs. 835.5 ± 45.2 (*p* < 0.001) (Fig. 2C).

These results can be confirmed by looking at various single tasks, regardless of level of experience. Table 1 shows the performance time for each task with regard to the different level of experience and gender.

The only exception are male NBCS in tasks “paper cut”, “recapping”, and “circle cutting”, as well as female BCS in tasks “needle threading”, “recapping”, “circle cut”, and “knot tying” where no significant difference for using a 3D- compared to a 4K-vision system could be observed.

Overall rate of mistakes while using 3D-vision vs. 4K-vision system was for female 10.2 ± 1.1 vs. 13.3 ± 1.3 (*p* = 0.005) and for male participants 9.6 ± 0.7 vs.12.2 ± 1 (*p* = 0.001) regardless of level of experience. For male MS it was 8.7 ± 1.2 vs. 13.8 ± 1.5 (*p* < 0.001) and for female NBCS 12.7 ± 1.2 vs. 17.3 ± 1.5 (*p* = 0.001). Among female MS, male NBCS as well as female and male BCS, no difference could be observed, as shown in Fig. 2.

### Benefit of female vs. benefit of male surgeons using 3D- vs. 4K-display system

**Fig. 3:**
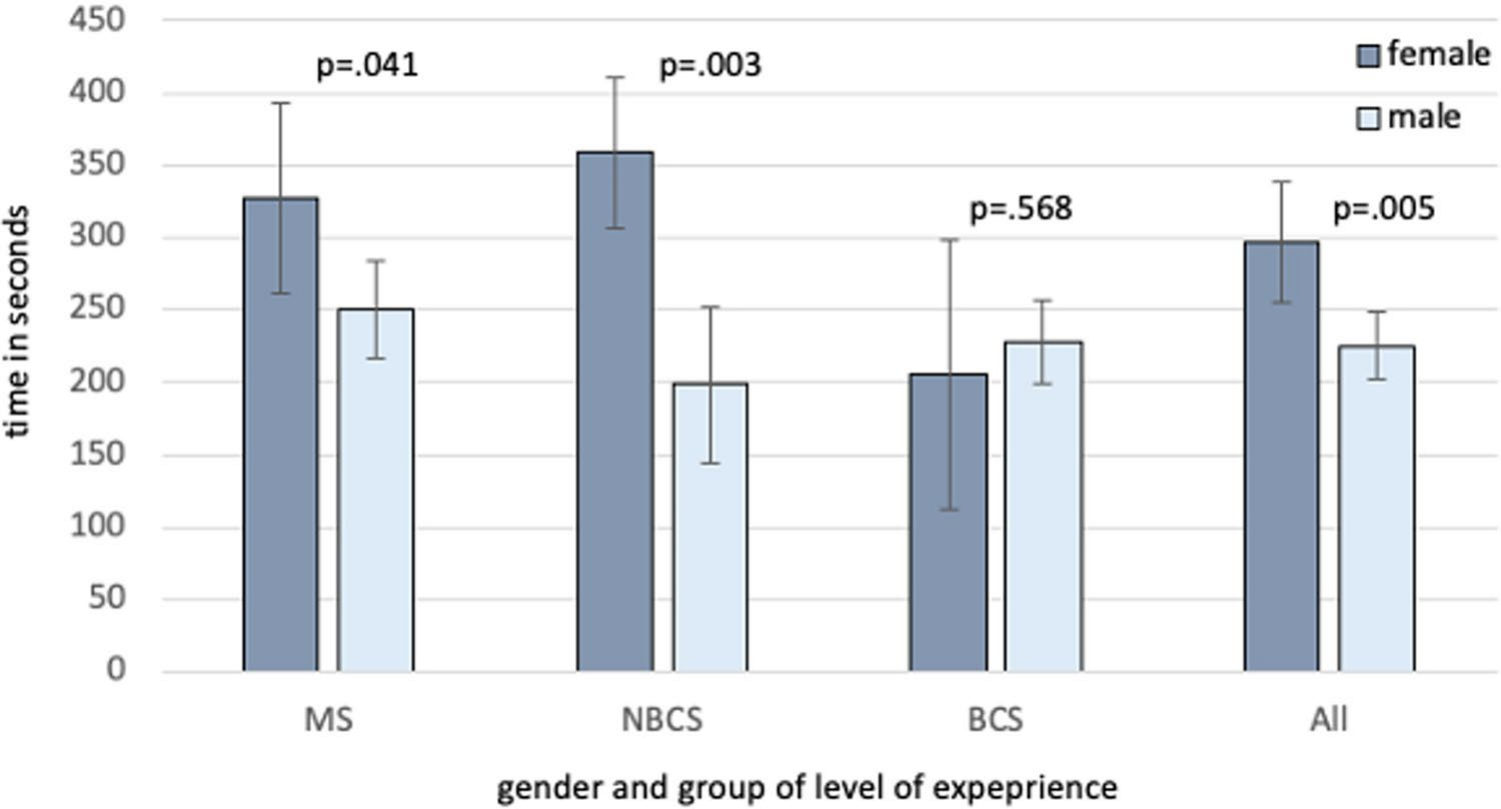
Benefit in performance time using 3D vs. 4 K comparing male and female participants. It shows the benefit in performance overall performance time (all tasks performed) in seconds when using a 3D-vision system compared to a 4 K-vision system at the minimally invasive training parkour—medicine students, non-board certified surgeons, and board certified surgeons comparing male and female participants. The benefit of using a 3D-vision system is more pronounced in female MS and NBCS, as well as for all females compared to their male colleagues. *MS* medicine students, *NBCS* non-board certified surgeons, *BCS* board certified surgeons, *p* *p* value, *3D* 3-dimensional-display system, *4 K* 2D-4 K ultra-high definition-display system

Regarding the differences between overall parkour time in 3D and 4K, in order to quantify the benefit of using a 3D-vision system compared to the 4K system, a comparison in between display systems among all female participants shows a difference of 297.3 ± 41.8 (improvement 27.8%) vs. 225.2 ± 23.3 s (improvement 25.3%) in men (*p* = 0.005), shown in Table 2. This can be confirmed in the MS group (327.6 ± 65.5 vs. 249.8 ± 33.7, *p* = 0.041) and in the NBCS group (359 ± 52.4 vs. 198.2 ± 54.2, *p* = 0.003). There was no significant difference in the BCS group, as shown in Fig. 3.

**Table 2:** Benefit in performance time 3D- vs. 4 K-display system

		Rope pass	Paper cut	Peg transfer	Needle threading	Needle recapping	Circle cutting	Knot tying	Overall parkourtime
All (n = 127)	w (*n* = 56)	28.9 ± 4.0	32.5 ± 9.9	33.9 ± 4.8	126.4 ± 21.8	35.8 ± 14.9	10.9 ± 7.7	71.8 ± 24	297.32 ± 41.8
	m (*n* = 71)	22.5 ± 2.1	16.6 ± 5.5	39.3 ± 2.6	85.8 ± 11.8	27.5 ± 8.5	17.2 ± 4.1	80.6 ± 13.2	225.15 ± 23.3
	p	0.032	0.044	0.127	0.016	0.483	0.278	0.671	0.005
MS (n = 48)	w (*n* = 21)	43.6 ± 5.4	44.4 ± 15.6	49.4 ± 6.5	163.3 ± 30.8	49 ± 20.5	ND	ND	327.6 ± 65.5
	m (*n* = 27)	42.6 ± 3.0	29.3 ± 8.1	56.1 ± 3.8	100.3 ± 17.5	50.3 ± 12.5	ND	ND	249.8 ± 33.7
	p	0.817	0.216	0.176	0.008	0.940			0.041
NBCS (n = 39)	w (*n* = 28)	23.1 ± 4.7	29.6 ± 11.1	29.2 ± 5.7	116.3 ± 26.4	41.4 ± 17.8	14.6 ± 6.8	132 ± 22.6	359 ± 52.4
	m (*n* = 11)	11 ± 4.7	4.7 ± 12.3	35.2 ± 6.0	104.0 ± 26.6	3.4 ± 19.3	7.7 ± 7.1	82 ± 23.2	198.2 ± 54.2
	p	0.011	0.004	0.302	0.645	0.042	0.321	0.030	0.003
BCS (n = 40)	w (*n* = 7)	20.1 ± 9.5	23.5 ± 22.8	23.1 ± 11.4	99.4 ± 51.4	16.9 ± 35.5	7.1 ± 13.8	11.6 ± 42.3	205.3 ± 93.4
	m (*n* = 33)	13.9 ± 2.7	25.3 ± 7.2	26.6 ± 3.5	53.1 ± 15.5	28.7 ± 11.1	26.7 ± 4.1	79.1 ± 12.7	227.4 ± 28.8
	p	0.329	0.912	0.636	0.190	0.642	0.036	0.051	0.568

It shows the benefit in performance in seconds time using a 3D-display compared to a 4 K-display system for each task of the minimally invasive training parkour for female and male medicine students, non-board certified surgeons, and board certified surgeons

*f* Female, *m* male, *MS* medicine students, *NBCS* non-board certified surgeons, *BCS* board certified surgeons, *ND* not done according to study protocol, *p* *p* value

Regarding the differences between overall mistakes in 3D and 4K, a comparison among the female NBCS shows a difference of 4.7 ± 1.4 vs. 1.4 ± 1.8 among men (*p* = 0.038). There was no significant difference, neither in the MS nor in BCS group, nor among all participants.

## Discussion

It is known that men and women are processing visual impressions in different ways [11–14, 16]. While it seems easier for women to interact between analytical and intuitive impressions and memorize words and faces, men tend to show fewer difficulties in connecting perception and coordinated actions [10, 13] and come with evolutionary better visual acuity [10, 15, 35]. It is not clear if these gender specific differences in visual image processing influence the performance in minimally invasive surgery of female and male surgeons. Data comparing laparoscopic performance between genders and gender specific benefits is currently sparse and inconsistent. Aim of this trial was to evaluate in a randomized cross-over trial, if there is a gender specific benefit using a 3D-display system in comparison to a 2D-4K-display system.

The EAES consensus of 2018 recommended, after reviewing 138 articles, the use of a 3D-vision system in laparoscopy to reduce operation time [36].

Wagner et al. compared a 3D- vs. 2D-display system with 34 participants of which 14 were female [37]. In a trial by Alaraimi et al. there were 23 out of 50 participants female [32]. Abdelrahman et al. compared a 3D- vs. 4K-display system with 15 women and 9 men [8]. All of them showed the advantage of using a 3D system, but analysis separated by gender did not take place in any of the studies mentioned. Additionally, the number of participants was constantly small, there was an insufficient spectrum of participants related to different levels of experience or the level of evidence regarding study design was low.

The main finding, regarding the primary outcome parameter, of our study was that both, female and male surgeons, benefit from using a 3D-display system compared to a high-resolution 2D-4K-display system. But female surgeons have a significant higher benefit using a 3D system compared to their male colleagues, referring to the secondary outcome parameter. Both groups could significantly decrease their overall parkour time using a 3D system: for all women from 1068.1 in 4K to 770.7 in 3D seconds by 28% and for all men from 889.7 s in 4K to 664.5 s in 3D by 25%.

This not only proves that both genders benefit from 3D, but also shows that the effect is more pronounced in female surgeons. This is most evident in surgically unexperienced groups of MS, with women performing 327.6 s faster by using a 3D-vision system (35.8%) compared to 249.8 s (32.12%) in men (*p* = 0.041) and NBCS women improved by 359 s (28.25%) vs. 198.2 s (18.62%) in men (*p* = 0.0003).

In experienced BCS with many years of training in open and minimally invasive surgery, men and women benefit equally.

This could be explained by behavioral science data: men find it neurophysiologically easier to deal with tasks that require 3-dimensional thinking as well as spatial processing because of better spatial imagination and orientation, while female brains tend to promote communication between processing of analytical and intuitive impressions [10, 13, 19, 20, 26, 38, 39].

Neurophysiological basic research by Ingalhalikar et al. supports this theory. They used diffusion tensor imaging of the brain to show the structural connectome. In their study, including 949 young people (428 males, 521 females), they could demonstrate that males had greater within-hemispheric connectivity in all supratentorial regions, whereas between-hemispheric connectivity and cross-module participation predominated in women. This suggests that male brains are structured to facilitate connectivity between perception and coordinated action, whereas female brains are designed to facilitate communication between analytical and intuitive processing modes [13].

Astur et al. had 20 male and 20 female participants perform a virtual Morris Water Task, where subjects needed to use the spatial arrangement of cues outside of a circular pool to swim to a hidden goal platform, to evaluate virtual place learning and navigation ability. They found males navigated to the hidden platform better than females across a variety of measures [19].

The abilities of males and females to make spatial inferences were compared by Ruggiero et al., where participants had to study line drawings and had to remember line distances and showed that males outperformed females in spatial inference and mental rotation [40].

In conclusion, it seems reasonable to assume that men can better imagine 3-dimensional objects and 3D representation is not so much needed. Men do not rely on a 3D-visual-system as much as women do and the disadvantage of a non-3D-display is noticeable to a lesser extent in men [13, 19, 20, 40, 41].

Our results do confirm this hypothesis by showing a more pronounced benefit of using a 3D-vision system in surgical unexperienced women.

This result is supported by the observations made by Donnon et al.. 42 medical students had to aquire laparoscopic suturing skills. Women initially showed less technical instrument handling ability than men. With more practice they catched up [39]. This might be an explanation for the fact that women benefit more initially, but this effect seems to be balanced out with increasing level of experience. Hence, there is no gender specific benefit difference in experienced surgeons after their formal surgical training.

It can be assumed that men and women still have different education in early childhood with girls having less contact with technology or computers [42]. Su et al. showed that men prefer working with things and women prefer working with people [43].

Recent studies underlined this, showing that gaming skills may be an advantage when learning laparoscopic surgery [44]. Gradl et al. put forward the hypothesis that women have less opportunity to deal with visual-spatial tasks in their childhood development and socialization, and conclude this may prove disadvantageous [45]. Transferred to laparoscopic surgical socialization, training and education this might be reflected by the initial higher benefit for female surgeons of using a 3D-vision system followed by the flattening curve with gaining level of experience due to the training effect.

Regarding differences in error rate of both systems, there was only a significant difference among the female NBCS with 4.7 vs. 1.4. The effect is therefore significantly more pronounced in terms of time compared to the errors. This also corresponds to the results that we were able to observe regardless of gender.

The number of female medical students is steadily increasing [1–3] and although proportionally fewer women than men are choosing a career in surgery, the number of female surgeons is increasing, too [2, 4, 5].

A balanced male–female ratio would not only be desirable in terms of equality, but would also help to improve the atmosphere during the operation, since men and women often show different soft skills [46].

McKinley et al. were able to observe that women showed significantly better impulse control during an operation, while men presented significantly better stress management [47]. Obviously, an operation team would benefit from both skills. It is conceivable this could improve the atmosphere, the surgical procedure and, ultimately, the patient's outcome.

One limitation is, that there is still no ophthalmological standard test for simulated stereoscopic vision as is used in the operation theater with passive polarizing 3Dsystems.

Another limitation is that percentage of women and men in the three groups of experience levels was different. This was balanced by the statistical methods used for the analysis, but it also reflects the real worlds issue with less female surgeons.

The difference we could show in this trial supports data of current research, showing that curricular training in laparoscopic surgery should be more flexible and may reduce differences between genders. As previous studies could demonstrate the use of a 3D-vision system should be considered, especially during training phase, to facilitate surgical apprenticeship [8, 32, 36, 48–51]. Our results can confirm this and emphasize the recommendation in particular for female surgeons. As Beattie et al. have shown, it remains questionable whether a 3D system based training can also improve surgical performance in 2D [52]. Further studies are required with regard to gender specific differences.

We do not believe that this allows a statement to be made about how good a surgeon is, as many other factors play an important role in the surgical work.

Yet we think in order to reduce the weaknesses of the respective gender and further promote their strengths, it is important to firstly recognize the difference.

In conclusion, women do benefit more from using a 3D-vision system during the training phase in minimally invasive surgery. After a high level of surgical experience has been reached, the benefit of 3D vision is equal for both, female and male surgeons.
